# Application of Fat-Tailed Sheep Tail and Backfat to Develop Novel Warthog Cabanossi with Distinct Sensory Attributes

**DOI:** 10.3390/foods9121822

**Published:** 2020-12-08

**Authors:** Leo Nyikadzino Mahachi, Monlee Rudman, Elodie Arnaud, Voster Muchenje, Louwrens Christiaan Hoffman

**Affiliations:** 1 Department of Livestock and Pasture Science, University of Fort Hare, Private Bag X 1314, Alice 5700, South Africa; leonyikadzinomahachi@gmail.com or vmuchenje@ufh.ac.za (V.M.); 2 Department of Animal Sciences, Stellenbosch University, Private Bag X1, Matieland, Stellenbosch 7602, South Africa; diehamsandwich@gmail.com (M.R.); elodie.arnaud@cirad.fr (E.A.); 3 CIRAD, UMR QualiSud, Matieland, Stellenbosch 7602, South Africa; 4 Qualisud, Univ Montpellier, Avignon Université, CIRAD, Institut Agro, Université de La Réunion, 97715 Montpellier, France; 5 Centre for Nutrition and Food Sciences, Queensland Alliance for Agriculture and Food Innovation (QAAFI), Agricultural Mechanisation Building A 8115, Office 110, Gatton, QL 4343, Australia

**Keywords:** consumer acceptance, fatty acids, lipid oxidation, physicochemical attributes, sensory attributes, venison

## Abstract

This study compared the use of pork backfat (PF) and fat-tailed sheep tail and backfat (SF) on the physicochemical, fatty acids and sensory attributes of warthog cabanossi. There were no differences between weight loss during drying, moisture content, pH, water activity, salt content and lipid oxidation between the cabanossi types. However, protein and ash contents were higher in PF cabanossi whilst fat content was higher in SF cabanossi. The PF cabanossi had higher polyunsaturated fatty acids (especially n-6), lower monounsaturated fatty acids whilst the saturated fatty acid content was similar between the two cabanossi products. The n-3:n-6 ratio was more beneficial in the SF cabanossi. The descriptive sensory analysis showed two distinct products where PF cabanossi scored higher for most attributes. Although SF cabanossi scored less for these attributes, this cabanossi had unique and acceptable sensory attributes. This study concluded that fat-tailed sheep tail and backfat could be used to produce a unique cabanossi product of acceptable quality.

## 1. Introduction

The increasing awareness of the deleterious health effects of fat and saturated fatty acids (SFA) composition of meat and meat products has contributed to advances in alteration of the fatty acid (FA) profile of these food commodities [1,2]. Modern consumers prefer lean meat with high levels of polyunsaturated fatty acids (PUFA), especially n-3 and n-6 FA. In the porcine industry, positive strides have been made to produce lean pigs that have a favourably higher content of PUFA in their meat. One of the methods employed to improve the FA profile of pork is appropriate feeding with feed containing favourable fatty acids [2,3,4,5,6,7].

Although high PUFA levels are beneficial, they are responsible for producing soft fat associated with a low melting point [8,9,10]. This negatively affects the processing properties, oxidative stability and shelf life of meat products [11,12,13]. Meat products containing soft fat consequently have reduced firmness, exhibit fat caps (fatting out) and have an oily/shiny appearance when packed [5,12,14] although different recipes, packaging and storage may also influence product quality [15,16]. Sensory attributes such as flavour and aroma are also negatively affected due to increased lipid oxidation of products developed using soft fat [12,13,17] especially in the case of salted/dried products such as cabanossi due to the pro-oxidant effect of salt [18]. Moreover, the processing of dry sausages manufactured with soft fat may be challenging as they may not achieve an adequate drying due to the liquefaction of fat which coats the lean particles [19,20]. Therefore, it is important to explore alternative fat sources which could potentially improve product quality.

Fat-tailed sheep reserve fat in their tails to be used during times when natural food resources are scarce [8,21]. However, management practices such as tail docking result in a shift of fat deposition site from the tail to muscle and subcutaneous tissues, and the latter increases backfat thickness [22,23,24]. Although fat derived from ruminants is predominantly SFA [2,4,9], feed restriction has been associated with improved fatty acid profile of sheep backfat [25,26]. Alves et al. [23] demonstrated that backfat from Damara (a fat-tailed sheep breed) under dietary restrictions, had 1.6% less palmitic acid (C16:0) mainly due to constrained de novo synthesis of lipids. On the other hand, the concentration of oleic acid (C18:1cis-9) increased due to stearoyl-CoA desaturase and lipogenic activity that occurs during backfat mobilization [25]. Moreover, van Harten et al. [24] observed higher levels of omega-3 FA, i.e., eicosapentaenoic acid (EPA; C20:5n−3), docosapentaenoic acid (DPA; 22:5n−3) and docosahexaenoic acid (DHA; C22:6n−3) in lipids from feed restricted Damara compared to non-fat-tailed sheep (Dorper and Australian Merino). Dietary manipulation is well known to improve the fatty acid profiles of sheep lipids [2,4,27]. Overall, lipids obtained from either fat-tailed sheep [8,26] or sheep fed PUFA-manipulated diets [2,3,4] have healthier FA profiles and could be valuable resources for developing meat products.

In various countries where the Muslim faith is the predominant faith and religious practices do not permit utility of pork in processed Halal foods, meat and fatty tissues from sheep and goats, including tail fat from fat-tailed sheep breeds are used to produce traditional and modern meat products [28,29,30]. In Iran, fat from fat-tailed sheep is used for cooking [8], whereas it is used to produce a variety of meat products in North Africa and Mediterranean regions [28]. Sheep fat has previously been used to produce beef-fermented sausages (Bez sucuk) [28,30] and is typically used to make droëwors (a typical South African dried sausage) [31] although its use in game meat products is still limited. While local people consider game meat as indigenous, it is also considered exotic and is attractive to adventurous consumers, particularly tourists, who want to experience new culinary experiences and take home products as souvenirs [32,33].

In South Africa, consumers enjoy indigenous meat products including droëwors, biltong (dried meat) and exotic meat products including salami (semi-dry fermented sausage) and cabanossi [33,34]. In that regard, some local artisanal manufacturers are acquainted with producing salami and cabanossi using game meat [35,36]. Cabanossi, alternatively kabanosy [33,37], is a semi-dry, cured and smoked pork sausage originating from Poland but has become a well-known snack among the South African population [33,36]. As of 2012, kabanosy was recognised by the European Union as a “Traditional Specialities Guaranteed” (TSG) product under the Commission Implementing Regulation No: 1044/2011 [38]. This registration, however, does not limit other producers from manufacturing the product although they may not use the same name if the recipe deviates from the original [36]. Traditionally, cabanossi was produced from a young fat pig (kabenek) without the addition of fat as opposed to the current practice of adding different fat sources to improve sensory attributes [36,39]. Nowadays, cabanossi is produced from several meat sources with additional fat for improved sensory attributes. Cabanossi produced from warthog meat and pork backfat (PF) was reported to be acceptable although criticized for its unhealthy FA profile [40], despite the favourable FA profile recorded for warthog meat [40,41], thereby stimulating research on other healthier fat sources. The aim of the current investigation was to determine the influence of fat-tailed sheep tail- and backfat (SF) on the physicochemical, fatty acids, lipid oxidation and sensory properties of warthog cabanossi.

## 2. Materials and Methods

### 2.1. Harvesting and Meat Sampling

Prior to the commencement of this study, ethical clearance was sought from the University of Fort Hare Research Ethics Committees (Ethical Clearance number: MUC361SMAH01). Warthogs (n = 24) used for meat in this study were harvested at a game farm (27° 22′ 09.26″ S, 31° 50′ 42.16″ E) near Pongola, KwaZulu Natal, South Africa. The farm falls within the Savannah biome, which is characterised by summer rainfall, mean annual precipitation ranging from 500–900 mm. The procedures for harvesting warthogs were described previously by Rudman et al. [42] Meat from entire warthog carcasses was used for cabanossi production except for the *m. longissimus thoracis et lumborum* muscle which was used by Rudman et al. [42] The SF used was sourced from pasture-fed Damara sheep while different batches of PF were purchased from a local abattoir in the Western Cape Province of South Africa. After harvesting and slaughter, meat and fat were vacuum packaged and stored at −20 °C until use.

### 2.2. Preparation of Cabanossi

The preparation of batter used to make cabanossi was similar to the method outlined previously [36]. Briefly, frozen warthog meat, PF and SF were thawed at <4 °C for ~12 h before use. Twelve separate 3 kg batches of PF (20%) and twelve separate 3 kg batches of SF (20%) cabanossi were produced. To avoid pseudoreplication, each batch of cabanossi was produced using meat from a unique animal (n = 24). To control the ratio between meat and fat in the batter, meat was trimmed of visible excess fat and sinews. Meat and fat were cut into ~5 × 5 cm cubes. Meat (2.4 kg) was weighed into a separate tray where 0.6 kg fat was added and hand mixed. After mixing, the mixture was minced through a 5 mm grinding plate (Manica, Model number CM-21, Equipamientos Carnicos, Barcelona, Spain) where after the spice mixture was added. The spice mixture consisted of 2% salt, 0.24% curing agent (Prague powder #1: Freddy Hirsh, Somerset West, South Africa), 0.2% black pepper, 0.06% nutmeg, 0.1% roasted and grounded caraway seeds and 0.2% mustard. The batter was mixed and minced again through the 5 mm plate before stuffing into 22 mm diameter sheep casings using a manual sausage filler (MOD. 7/V, Tre Spade, Torino, Italy). The cabanossi were hung in a temperature and humidity controlled drying chamber (Reich Airmaster^®^ UKF 2000 BE, Reich Klima-Räuchertechnik, Urbach, Germany) for 16 h as described previously [33].

### 2.3. Physicochemical Analyses

The weight loss of each cabanossi batch was calculated as the percentage of lost weight relative to the initial weight of that batch. The pH of the raw batter was measured using a Crison 25 pH meter (Crison Instruments S.A., Alella, Spain) with an electrode probe. To determine the pH of the finished product, 3 g of sample was homogenised in 27 g dH_2_O in duplicate before pH was measured using a Crison 25 pH meter with an electrode probe. Water activity was measured in duplicate using an Aqua Lab Due Point and Water Activity Meter 4TE (Decagon Devices, Inc., Pullman, WA, USA) at 25 °C. Moisture and ash were analysed according to the procedures of AOAC [43]. Fat was extracted using a chloroform/methanol (2:1 *v*/*v*) solution and determined according to Lee et al. [44] The defatted and dried meat samples (dried for at least 48 h at 60 °C in an oven) were analysed for nitrogen [43] using a calibrated LECO Nitrogen/Protein analyser (FP-528, Leco Corporation, St. Joseph, MI, USA). Protein was then calculated by multiplying the percentage of nitrogen by 6.25. All proximate analyses were performed per batch, in duplicate. Salt content was determined in duplicate by analysing the chloride concentration of each sample using a chloride analyser (Model 926, Sherwood Scientific, Cambridge, UK) after extraction from 0.3 g of sample in 50 mL 0.3 M nitric acid for at least 2 h.

### 2.4. Fatty Acid Composition

Fatty acid composition was determined by gas chromatography after extraction of lipids in chloroform/methanol (2:1 *v*/*v*) and transmethylation in methanol/sulphuric acid (19:1 *v*/*v*) as described by Neethling et al. [45] Fatty acid composition was expressed as a percentage of the content in the sample of total fatty acids. To assess the nutritional properties of the cabanossi, the ratios PUFA:SFA and n-6:n-3 were calculated. Lipid health indices (atherogenicity; AI and thrombogenicity; TI) of Ulbricht and Southgate [46] were also determined.

### 2.5. Lipid Oxidation

Lipid oxidation was determined by measuring thiobarbituric acid reactive substances (TBARS) on the raw batter and the finished cabanossi product using an acid-precipitation method previously described by members in our research group [47]. Absorbance was measured at 530 nm (Spectrostar Nano, BMG Labtech, Ortenberg, Germany) and TBARS were expressed as malondialdehyde (MDA) equivalent content (mg/kg).

### 2.6. Descriptive Sensory Analysis

Twelve replicates (n = 12) of each of the two cabanossi treatments were subjected to descriptive sensory analysis (DSA) by a panel of 12 judges trained according to approved procedures [48]. During training, the panellists made use of specific reference samples to formulate a list of sensory attributes in the order of aroma, flavour, appearance and texture (Table 1). The attributes for the descriptors were scored on an unstructured scale from 0–100. To cleanse the pallet between samples, panellists were given fresh apple quarters, water biscuits and spring water stored at room temperature (21 °C). After training, blind testing of the products was done over 12 replicate sessions that lasted six days. The judges sat in individual booths in a temperature (21 °C) and artificial daylight-controlled room. Each booth had a computer on which Compusense^®^ five software (Compusense, Guelph, Canada) was installed.

### 2.7. Consumer Preference

The cabanossi were evaluated for preference to taste and appearance by 131 untrained consumers following a methodology described by Mahachi et al. [36] Each consumer was given two samples to evaluate; one from each treatment in the company of a corresponding questionnaire to fill and rate their preference for each sample. The questionnaire asked for demographic information and provided an unstructured scoring scale from 1–9 on which they could rate their preference for the different cabanossi treatments. All consumer identifiers were scrubbed from the data before analyses. The demographic information of the sample population is shown in Table 2.

### 2.8. Statistical Analysis

Physicochemical analyses data were statistically analysed using the generalised linear model procedures of SAS software (Version 9.4; SAS Institute Inc., Cary, NC, USA) in a completely randomised block design with the fat type as the main effect. Observations over time were combined in a split-plot analysis of variance with production stage (raw batter and finished product) as a sub-plot factor. For DSA data, judges (n = 12) were considered as block replicates for each sample (backfat type × replicate). A Shapiro–Wilk test was performed on the standardised residuals from the model to test for normality. In cases where there was significant deviation from normality, outliers were removed when the standardised residual for an observation deviated with more than three standard deviations from the model value. The data for consumer acceptance were analysed using mixed model repeated measures of ANOVA. Fisher’s least significant difference was calculated at the 5% significance level to compare treatment means. 

## 3. Results and Discussion

### 3.1. Physicochemical Attributes

Results for the physicochemical analyses are presented in Table 3. There were no significant differences in weight loss during drying between treatments while moisture loss was higher (*p* ≤ 0.05) for the PF treatment compared to the SF treatment. Moisture content was higher (*p* ≤ 0.01) in the raw batter of the PF treatment compared to the SF treatment. This phenomenon may be explained by the fact that there were differences (*p* ≤ 0.01) in the chemically analysed fat content of the two treatments’ raw batter. Although similar amounts of fat were added during the mixing of batter, the sheep fat raw batter had more analysed fat compared to the pork fat treatment (*p* ≤ 0.01). The suggestion would be that sheep subcutaneous and tail adipose tissue used in this study had more lipids and thus less moisture per unit of weight compared to that of pork. This could be attributed to de novo synthesis of lipids and fatty acids in sheep adipose tissue. Ruminant diets have a low fat content and hence most of their lipids and fatty acids are synthesised de novo in adipose tissue [22,49]. As a result, cabanossi end products showed a similar moisture content (*p* > 0.05). The lower moisture loss percentage of SF cabanossi could be related to saturation of sheep fat [50] or the fact that the fat content of PF raw batter was lower than that of the SF raw batter. Fat reduces water losses during drying of meat products by forming an oily coating around meat particles henceforth acting as “insulation” [36], which consequently limits the diffusion of moisture from the inside-out of the sausage [37,50].

Moisture content was within the range (39–50.7%) reported for commercial cabanossi [37,51], but lower than that reported by other authors for warthog and pork cabanossi (59% and 54%, respectively) [33]. Results from this study were also comparable to those reported in our companion paper (45.6%) [36] for warthog cabanossi produced with 20% PF, a similar amount as that used in the current investigation. Cabanossi were dried under temperatures that are not lethal to microorganisms, thus, like other meat products falling into this category, it should rely on the collective effects of safety hurdles of reduced water activity, pH and curing salts to prevent microbial spoilage [52,53]. These hurdles must be achieved in the order of: water activity <0.91 or pH < 4.5 or a combination of water activity <0.95 and pH < 5.2 [54]. These hurdles were achieved. Water activity was reduced in the final products but did not differ (*p* > 0.05) between treatments. Apart from the water activity we reported previously [36], which is comparable to the current study, no other literature was found reporting the water activity of cabanossi. This could be due to limited studies on cabanossi reported in the English language since this is originally a Polish product. Regarding pH, no differences were observed for raw batter and the finished product between treatments. Nevertheless, pH declined significantly (*p* ≤ 0.001) after smoking and drying. The pH values obtained in this study are slightly higher than those observed for fermented sausages [55,56] where it is reported to fall below 5.0. This is expected because cabanossi is not fermented, i.e., no starter culture and/or sugars are added. In fermented sausages, pH declines as a function of increasing organic acids produced by predominantly lactic acid bacteria population growth from the starter cultures [57,58]. The pH of fermented meat sausages is expected to decline during drying and this allows it to reduce the rate of microbial spoilage [58]. Although not measured, the pH decline observed in this study could be attributed to the organic acid compounds of smoke onto the product during smoking [54].

Ash content in PF cabanossi was higher than in SF cabanossi while they were similar in the raw batter, which could be attributed to higher weight loss (although not significant) and moisture loss (*p* ≤ 0.01). Additionally, the higher levels of total ash of the cabanossi end products (when compared to raw meat) are expected because the addition of salt and spices to raw batter increases the ash content [59]. Protein content was higher (*p* ≤ 0.05) in PF cabanossi than sheep cabanossi as it was in the raw batter (*p* ≤ 0.05). This is due to the fat:protein ratio being lower in low fat sausages as opposed to high fat sausages as previously reported [60].

### 3.2. Fatty Acid Composition

Table 4 shows results for the fatty acid composition of PF and SF cabanossi. The two most abundant SFA in the cabanossi products were palmitic acid (~22%) and stearic acid (~14%) and these fatty acids were similar in concentrations for both products. Similarly, the percentage total SFA did not differ between treatments. Total MUFA was higher and total PUFA lower in SF cabanossi compared to PF cabanossi (*p* < 0.0001) whereas the PUFA:SFA ratio was lower in the sheep cabanossi (*p* < 0.0001). The PUFA:SFA ratio is an important indicator of the healthiness of meat products and dietary guidelines suggest a ratio of no less than 0.4 [61]. Results from this study revealed that PF cabanossi could be beneficial in this regard. Linoleic acid was the most abundant PUFA in both treatments, though it was higher in PF cabanossi compared to the SF treatment (*p* < 0.0001). The abundance of linoleic acid in the pork backfat treatment is attributed to its occurrence in pork backfat [6,7,62], and warthog meat [40,41] where it is reported to be the most abundant PUFA.

Omega-6 fatty acids were notably higher in the PF cabanossi and there were no differences in the n-3 fatty acids between treatments whereas the n-6:n-3 ratio was lower (*p* < 0.0001) in the SF cabanossi. The n-6:n-3 ratio is also thought to be a good indicator of meat healthfulness [2,63]. It is recommended that healthy meat products should exhibit a ratio of less than 4.0 [64], therefore the SF cabanossi (2.65) produced in this study could be beneficial. A high n-6:n-3 ratio is linked to pathogenesis of some illnesses including certain cancers and some inflammatory and cardiovascular diseases while a lower ratio reduces the incidence of these ailments [65,66]. However, with regards to cabanossi, this may be less important because it is mostly consumed from time to time in limited quantities as a snack rather than regularly as food [36]. Other important indicators of the healthfulness of meat are the atherogenic index (AI) and thrombogenic index (TI) of Ulbricht and Southgate [44], which are determined on the basis that different fatty acids metabolise differently, either preventing or promoting atherosclerosis and coronary thrombosis [46,67]. Although the AI was similar between the two treatments, the TI was lower in the PF cabanossi (*p* < 0.04). Results from this study present an opportunity to label both warthog cabanossi products as healthier than several other dry-cured beef and pork meat products whose AI and TI range between 0.50–0.67 and 1.09–1.45, respectively [65,67].

### 3.3. Lipid Oxidation

Results for lipid oxidation are shown in Table 3. No differences were observed in terms of the TBARS detected in the raw batter and cabanossi products. The TBARS of cabanossi reported in this study (0.35 and 0.37 mg MDA equivalent/kg) are lower than those reported previously [59] for ostrich droëwors (7.99 mg MDA equivalent/kg) where pork backfat was used while Mukumbo et al. [47,68] reported values in pork droëwors reaching 0.7–3.8 mg MDA equivalent/kg dry matter (0.6–2.9 mg MDA equivalent/kg) at the end of drying. Deriving from these results, the extent of lipid oxidation during the manufacture of warthog cabanossi using these fat sources can be considered minimal. However, due to the differences in MUFA, PUFA and PUFA:SFA, it would have been interesting to observe the shelf stability of these two products, but this could not be done due to limitations of this study, thus further research on this aspect is recommended.

### 3.4. Descriptive Sensory Analysis

The sensory profiles of the two cabanossi treatments are presented in Table 5. There were significant differences for all aroma attributes (*p* ≤ 0.01) except peppery aroma (*p* > 0.05). The PF treatment was scored higher for most of the aroma attributes. During the sensory panel training, some unique characteristics were detected in the SF cabanossi. Consequently, it was expected that this treatment would score higher for these characteristics viz., charred aroma, sheep-like fatty aroma, mutton aroma and herbaceous aroma (*p* ≤ 0.01). The presence of these sensory attributes could be as a result of mutton specific heterocyclic compounds such as 2-ethyl-3,6-dimethylpyrazine and 2-pentylpyridine, as well as branched chain volatile fatty acids (BCFA) such as 4-methlyphenol acid, 4-methylnonanoic acid and 3-methyl-indole acid, commonly known as skatole [69]. There is a strong link between these BCFA and mutton aromas and flavour [70,71,72]. In fact, 4-methlyphenol, 4-methylnonanoic and 3-methyl-indole are BCFAs thought to be precursors of undesirable rancid odour and flavour in mutton [70]. Although the origin of these compounds is not clearly understood, they could be products of rumen metabolism of pasture species in the sheep diet [69,70]. Skatole, however, is known to be one of the major compounds that cause boar taint in *Suidae* spp. [69], thus the absence of these sensory attributes in PF cabanossi could be a good indication of the absence of boar taint in both pork backfat and warthog meat. Therefore, the hypothesis that these sensory attributes are SF-related is strengthened.

Pork backfat cabanossi tasted saltier (*p* ≤ 0.05) than SF cabanossi although the salt content (~2.7%) did not differ (*p* > 0.05) in the chemical analysis (Table 3). This is attributed to the PF cabanossi being less fatty (chemically) with more protein. The bond between chloride ions and meat proteins is stronger than that of chloride and sodium ions (Hamm [73] cited in [74]), therefore, the extent to which chloride ions bind to protein may be strong enough to suppress the perception of salty flavour [74]. Regarding fatty mouthfeel, it was expected that SF cabanossi would score higher than PF cabanossi since it was higher in chemically analysed fat. Fat particles produce an oily coating around meat particles and this phenomenon causes a higher impression of fat upon chewing the cabanossi.

Concerning appearance, there were no differences (*p* > 0.05) in perceived percentage fat and fatty/oily/shininess, but the red-brown colour intensity differed between the two treatments. Red brown colour intensity was higher in PF cabanossi compared to SF cabanossi. This may be the influence of more protein recorded for PF cabanossi as opposed to more fat in the SF cabanossi. Fat is usually lighter in colour, thus, if more of it is present in a product, it will mask some of the dark/red colour of meat proteins.

Texture attributes were all significantly different between the two cabanossi products. Pork backfat cabanossi scored higher for first bite, chewiness and residue, whereas this was not the case for sustained juiciness. This is attributed to differences in protein and fat content of the products. Fat reduces hardness of meat and meat products by facilitating the diffusion of moisture during biting and mastication of meat [75]. Less energy and force are therefore required to successfully chew the product resulting in less residue. Furthermore, high fat meat sausages exhibit a better impression of juiciness compared to low fat sausages [75].

**Figure 1:**
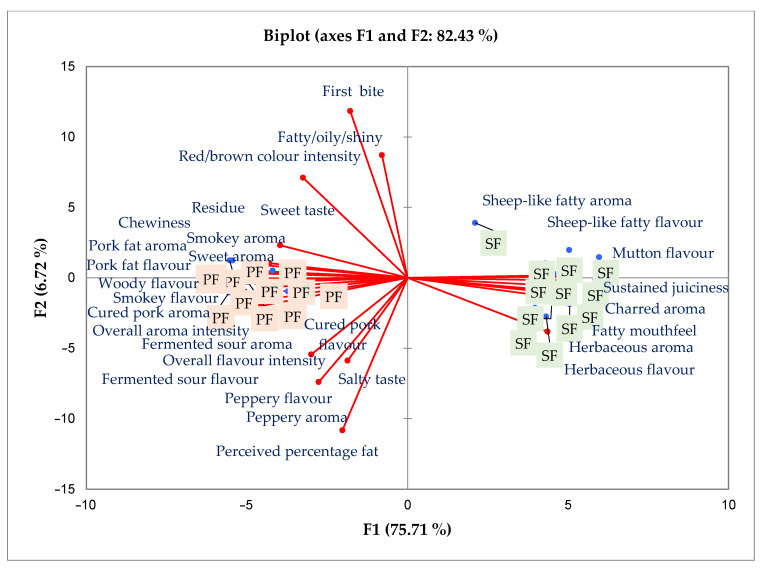
Principal component analysis for the sensory attributes of cabanossi made with either pork backfat (PF) or fat-tailed sheep fat (SF).

Figure 1 is a principal component analysis (PCA) showing the variation and grouping between the two cabanossi products. Factor 1 accounted for the most variation between the cabanossi. The PCA shows a clear distinction between the two products, with each being associated with specific attributes. The SF cabanossi was more associated with sheep-like fatty aroma and flavour, mutton fat aroma and flavour, herbaceous aroma and flavour, charred aroma as well as sustained juiciness. On the other hand, PF cabanossi was more associated with various aroma and flavour attributes typically linked to pork products as shown on the PCA.

### 3.5. Consumer Preference

**Figure 2:**
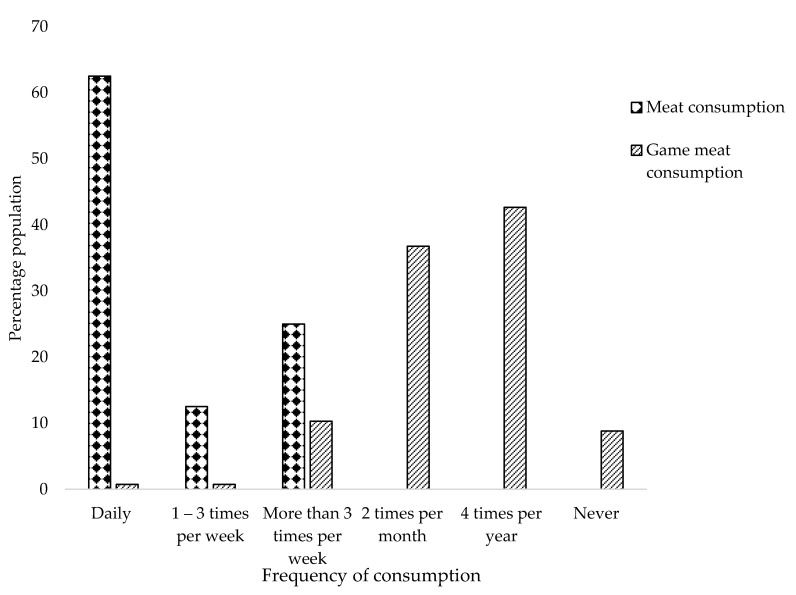
Meat consumption behaviour within the sample population.

In order to accurately predict consumer behaviour and attitudes towards new food products, it is important to understand various aspects of the population including their preference, choice, desire to eat certain foods, purchase intent and frequency of consumption [76]. Population demographic characteristics are a known influential factor on the sensory acceptance of healthier, reformulated meat products [76,77,78]. Results from the study revealed that most people consumed meat frequently (Figure 2). The majority of the population (62.5%) consume meat on a daily basis, whilst 25% consumed meat more than three times per week and only 12.5% of the population consumed meat between 1–3 times per week. However, game meat was not frequently consumed, with the majority (44.7%) of the population indicating that they only ate it approximately four times a year, whereas 36.8% of the population attest to consuming game meat at least twice a month. Whilst level of ethnicity and education were not included in the analyses due to statistical imbalances, gender and age group influenced (*p* > 0.05) the frequency of consumption of neither domestic nor game meat. Burger [79] reported that the consumption of game meat in North America was influenced by demographic characteristics such as ethnicity, gender and household income. Other studies such as that of Hoffman [32] also reported an association between ethnicity and game meat consumption in South Africa as some population groups associated it with leanness and healthiness, as well as it having a favourable gamey flavour which was not perceived as important by others. Game meat consumption may also be influenced by the lifestyles and social activities of different populations as families that are involved in hunting are more likely to regularly consume game meat [80]. However, a recent study did not report any associations between demography and game meat consumption [33].

Consumers were asked to rank their preference (on a scale of 1 to 9; the higher the number the more positive their preference) for various game meat products in addition to factors that influence their purchasing decisions for game meat products. Least significant means for these rankings are shown in Table 6. In the order of preference, biltong (rating of 7.7) was the most preferred product followed by droëwors (6.9), fresh meat (6.7) and fresh/raw sausage (6.6), salami (6.3) and cabanossi (6.3). These results indicate that game meat might be more preferred if marketed as processed meat products rather than fresh meat as supported by the literature [81]. Consumers perceive fresh game meat to be difficult to prepare [32,81], probably due to limited knowledge on preparation methods, and would therefore prefer to consume it processed.

Results from this study suggest that biltong was the most preferred game meat product among the sample population. This could be attributed to the fact that biltong has a strong linkage to the South African tradition as a meat preservation strategy that has long been known [35]. Furthermore, in South Africa, biltong is produced by small artisanal (e.g., households and butcheries) to large commercial manufactures [35], and thus most consumers who participated in the study could have developed a preference for this product at some point during their upbringing. Some of these consumers could have had experience in making biltong at home, whilst others got exposure to it in local butcheries and retail outlets. Therefore, background knowledge of a product could have effects on its acceptance as a desirable food. Henceforth, in the current investigation, it was interesting to note that contrary to the suggestions that consumers preferred processed game meat compared to fresh meat, consumers actually preferred fresh game meat compared to cabanossi and salami (Table 6). Since cabanossi and salami have Polish and Italian origins, respectively, they may not have been popular products among the sample population, which could have contributed to less preference. Consumer acceptance of different meat products is often a complex phenomenon encompassing several factors which include psychological and demographic factors as well as food choice habits [77]. Less preference reported for cabanossi and salami in this study could also be attributed to their high price since price was the single most important factor (7.2) affecting purchasing decisions (Table 6), especially if the product did not offer any known benefits to the consumer. Processed meat products are expected to fetch higher prices per kilogram unit because of a greater resource input. Therefore, these products must be produced to provide benefits beyond basic nutrition as functional meat products [82] if their preference among South African groups is to increase. The second most important factors determining the purchase of game meat products were fat content (6.5) and safety (6.7), while species (5.8) and origin (5.5) were ranked as the least contributing factors. Consumer trends are shifting towards eating healthy foods with reduced fat content to limit the onset of cardiovascular diseases, certain cancers and other food-related complications including microbial poisoning [82,83], although purchasing decisions for these meat products are governed by the availability of disposable income [77].

Least significant means for overall taste and appearance scores for the two-cabanossi treatments under current investigation are shown in Table 7. There were significant consumer preference differences (*p* ≤ 0.01) pertaining to taste and appearance between the two cabanossi treatments. The PF cabanossi was ranked higher for appearance followed by the SF treatment. The appearance of the product produces the first impression that consumers will judge it by. Consumers use this impression to estimate product freshness, quality and probable sensory characteristics [84]. The observation that PF cabanossi was more preferred in terms of appearance could be linked with higher ratings for red/brown colour intensity observed in the DSA. Consumers prefer darker, redder sausages with less perceived percentage fat because they consider them healthier meat products [85]. Although the perceived percentage fat was similar in both treatments during DSA, during consumer analysis, it was observed that SF cabanossi had an external oily sheen which could have been caused by pressure exerted on the sausages during vacuum packaging. Similarly, the observation that PF cabanossi scored higher for taste is in accordance with the DSA, which found that it received higher scores for the most favourable flavour attributes including overall flavour intensity, smoky flavour, cured pork flavour, peppery flavour and sweet taste (Table 5). Generally, both treatments were rendered acceptable by the consumers, receiving scores of more than 6. The consumers’ acceptance of cabanossi from this study suggests that it is possible to produce acceptable cabanossi using SF.

## 4. Conclusions

Utility of different types of fat affects some physicochemical and sensorial characteristics of meat products. The findings obtained from this study indicate that fat-tailed sheep tail and backfat can be used as an alternative to pork backfat without detrimental effects on the physicochemical characteristics of cabanossi. However, results from descriptive sensory analysis indicate that this replacement produces products that are far apart from each other concerning aroma, flavour and texture, although, according to the ratings within the scales used, both products are acceptable. Although PF cabanossi were scored higher for most sensory attributes, SF cabanossi had some unique pleasant sensory attributes that are acceptable to consumers. Therefore, fat-tailed sheep tail and backfat may be used not necessarily as a replacement for pork backfat, but to produce another variety of cabanossi to diversify consumer choices. It may be necessary, and therefore, recommended that a shelf-life study be conducted to determine the influence of SF on shelf stability and flavour compounds of cabanossi. Valorisation of fat-tailed sheep breeds fat to develop new meat products that may be useful in improving income for artisanal meat product manufactures through product diversification. However, this may be dependent on region/country as people from areas where eating pork is not acceptable may be more receptive of sheep meat aroma and flavour in their meat products.

## Figures and Tables

**Table 1:** Sensory attributes description, scale and reference standards used for the descriptive sensory analysis of two cabanossi treatments.

**AROMA 0 = None, 100 = Prominent**	**Description of Attributes**	**Description of References**
Overall aroma intensity	The overall intensity of aroma upon removing the cover	
Smokey aroma	Aroma associated with wood smoke	Low, moderate and high intensity liquid smoke solution ^a^
Charred aroma	Aroma associated with blackened sheep meat cooked over high heat/flames	Braaied (barbequed) sheep meat
Fermented sour aroma	Aroma associated with all fermented meat products	Black forest ham/salami sticks
Cured pork aroma	Aroma associated with cured pork products	Cured unsmoked pork product (Ham)
Pork fat aroma	Aroma associated with roasted pork fat	Fat from roasted pork loin ^b^
Mutton aroma	Aroma associated with mutton	Mutton chops ^b^
Sheep-like fatty aroma	Aroma associated with mutton fat	Mutton fat ^b^
Peppery aroma	Aroma associated with black pepper	Crushed black peppercorns
Sweet aroma	Sweet aroma associated with molasses	Low, moderate and high intensity molasses solution
Herbaceous aroma	General mixed herbs aroma	Rosemary, thyme, parsley and organum
**FLAVOUR 0 = None, 100 = Prominent**	**Description of Attributes**	**Description of References**
Overall flavour intensity	The overall intensity of flavour upon chewing	
Smokey flavour	Flavour associated with wood smoked pork	Fried bacon ^c^
Woody flavour	Flavour associated with the wood from smoking	Wood sawdust solution ^d^
Cured pork flavour	Flavour associated with cured pork products	Black forest ham/Fried bacon
Fermented sour flavour	Flavour associated with all fermented meat products	Black forest ham/Salami sticks
Pork fat flavour	Flavour associated with roasted pork fat	Roasted pork loin fat
Mutton flavour	Flavour associated with mutton	Mutton chops
Sheep-like fatty flavour	Flavour associated with mutton fat	Mutton fat
Peppery flavour	Flavour associated with black pepper	Crushed black peppercorns
Herbaceous flavour	General mixed herbs flavour	Rosemary, thyme, parsley and organum
Salty taste	Salty taste	Low, moderate, high intensity salt solution ^e^
Sweet taste	Sweet aroma associated with molasses	Salami sticks
Fatty mouthfeel 0 = low, 100 = extremely high	The amount of fatty coating left on palate after swallowing	
**APPEARANCE**	**Description of Attributes**	**Description of References**
Red/brown colour intensity 0 = light, 100 = dark	Red/brown colour associated with different meat products	Dry cabanossi ^f^
Fatty/Oily/Shininess 0 = dull, 100 = shiny	A measure of how oily the product looks (shininess)	Dry cabanossi/salami sticks
Percentage fat 0 = lean, 100 = fatty/abundant	The percentage perceived fat in the products	Dry cabanossi/pepper salami
**TEXTURE**	**Description of Attributes**	**Description of References**
First bite 0 = low, 100 = high	The amount of pressure required to bite through the cabanossi	Vienna’s/salami
Sustained juiciness 0 = dry, 100 = extremely juicy	The impression of juiciness after first 5 chews	
Chewiness 0 = soft, 100 = extremely hard	The ease of chewing	Vienna’s/salami sticks
Residue 0 = none, 100 = abundant	The amount of residue left in mouth after 10 chews	

^a^ Low, moderate and high intensity were made using 1, 2 and 3 drops of liquid smoke solution in 100 mL water; ^b^ Roasted pork/mutton loin was roasted to an internal temperature of 75 °C and surrounding fat was used; ^c^ Fried bacon, pan fried bacon; ^d^ Wood sawdust solution, a cup-full (250 mL) of pine wood saw dust was soaked in water overnight and filtered; ^e^ Low, moderate, high intensity salt solution was made using 0.2%, 0.5% and 1% salt solution; ^f^ Dry cabanossi, a batch of cabanossi produced with no additional fat.

**Table 2:** Demographic variation of the sample population according to gender and age group.

Gender	Proportion *	Age Group	Proportion *
Male	60	18–23	20
Female	40	24–29	28
		30–39	22
		40–49	18
		50+	12
Total	100		100

* Proportion expressed as a percentage of the total population size.

**Table 3:** Physicochemical attributes and lipid oxidation of raw batter and cabanossi made with either pork backfat (PF) or sheep tail/backfat (SF).

Treatment	Raw Batter		*p*-Value	Finished Cabanossi Product		*p*-Value
	PF	SF		PF	SF	
Weight (kg)	2.6 ± 0.14	2.8 ± 0.16	0.302	1.7 ± 0.14	1.8 ± 0.21	0.097
Weight loss (%)	-	-	-	35.9 ± 4.75	33.2 ± 5.16	0.067
Moisture loss (%)	-	-	-	33.0 ± 0.85	29.9 ± 0.67	0.031
Water activity	-	-	-	0.94 ± 0.01	0.94 ± 0.01	0.146
pH	5.58 ± 0.17	5.56 ± 0.14	0.563	5.16 ± 0.10	5.14 ± 0.11	0.578
Moisture (%)	63.1 ± 0.88	60.7 ± 1.39	<0.0001	46.0 ± 1.16	46.2 ± 2.16	0.364
Protein (%)	19.9 ± 3.80	16.7 ± 3.34	0.030	27.8 ± 3.70	25.9 ± 2.09	0.032
Fat (%)	16.2 ± 4.28	20.5 ± 3.50	0.001	23.2 ± 4.40	24.8 ± 2.91	0.027
Ash (%)	2.7 ± 0.12	2.7 ± 0.10	0.502	4.0 ± 0.26	3.8 ± 0.22	0.006
Salt (%)	1.9 ± 0.11	1.9 ± 0.10	0.688	2.8 ± 0.25	2.7 ± 0.14	0.744
TBARS (mg/kg)	0.21 ± 0.07	0.24 ± 0.06	0.259	0.35 ± 0.09	0.37 ± 0.08	0.390

All data expressed as mean ± SE (n = 12).

**Table 4:** Percentage fatty acid composition of cabanossi made with either pork backfat (PF) or sheep tail/backfat (SF) before and after drying.

* FATTY ACID	Raw Batter		*p*-Value	Finished Cabanossi Product		*p*-Value
	PF	SF		PF	SF	
***Saturated fatty acids***						
C14:0 Myristic	1.69 ± 0.19	2.42 ± 0.48	0.001	1.38 ± 0.31	1.37 ± 0.30	0.356
C16:0 Palmitic	22.8 ± 1.34	21.95 ± 2.24	0.062	21.90 ± 1.92	22.16 ± 1.38	0.902
C18:0 Stearic	14.56 ± 2.21	12.79 ± 3.02	0.001	14.33 ± 2.03	14.27 ± 2.04	0.908
***Monounsaturated fatty acids***						
C16:1 Palmitoleic	2.10 ± 0.37	3.96 ± 1.08	0.000	1.9 ± 0.21	2.0 ± 0.43	0.741
C18:1n9c Oleic	23.49 ± 2.16	32.57 ± 5.10	<0.0001	24.4 ± 2.99	33.8 ± 2.75	0.637
***Polyunsaturated fatty acids***						
C18:2n6c Linoleic	22.32 ± 1.91	8.27 ± 6.15	<0.0001	21.47 ± 5.60	5.24 ± 0.65	<0.0001
C18:3n3 γ-α-Linolenic	2.59 ± 0.61	2.24 ± 0.49	0.030	2.58 ± 0.63	2.40 ± 0.77	0.040
SFA	42.95 ± 3.35	42.30 ± 4.53	0.864	43.18 ± 4.57	43.73 ± 5.80	0.580
MUFA	28.38 ± 2.50	43.30 ± 8.57	<0.0001	29.27 ± 3.84	45.38 ± 5.68	<0.0001
PUFA	28.67 ± 1.88	13.90 ± 6.15	<0.0001	27.55 ± 6.00	10.88 ± 1.32	<0.0001
PUFA: SFA	0.67 ± 0.09	0.32 ± 0.13	<0.0001	0.65 ± 0.18	0.25 ± 0.05	<0.0001
Total n-6	25.35 ± 1.85	10.96 ± 6.12	<0.0001	24.28 ± 5.78	7.82 ± 0.74	<0.0001
Total n-3	3.32 ± 0.64	2.94 ± 0.51	0.033	3.27 ± 0.71	3.07 ± 0.79	0.361
n-6:n-3	7.88 ± 1.49	3.79 ± 2.11	0.0001	7.33 ± 2.25	2.65 ± 0.50	<0.0001
***Health indices***						
Atherogenic index	0.43 ± 0.02	0.59 ± 0.07	<0.0001	0.44 ± 0.06	0.62 ± 0.04	0.050
Thrombogenic index	0.87 ± 0.12	0.94 ± 0.18	0.118	0.90 ± 0.19	0.99 ± 0.23	0.040

* Fatty acids: individual fatty acids with a percentage composition less than 1 were not displayed on the table but were included in calculation of total SFA, MUFA, PUFA, n-6 and n-3; All data are expressed as mean ± SE (n = 12).

**Table 5:** Sensory attributes of cabanossi made with either pork backfat (PF) or sheep tail/backfat (SF).

AROMA	PF	SF	*p*-Value
Overall aroma intensity	66.28 ± 1.85	56.66 ± 2.28	0.002
Smoky aroma	63.47 ± 1.77	52.34 ± 2.64	0.001
Charred aroma	4.94 ± 1.65	16.75 ± 1.92	0.001
Fermented sour aroma	19.27 ± 1.59	13.86 ± 1.41	0.006
Cured pork aroma	25.94 ± 1.97	15.44 ± 1.62	0.000
Pork fat aroma	17.10 ± 2.00	4.40 ± 1.79	0.000
Mutton aroma	1.02 ± 0.96	8.68 ± 1.29	0.000
Sheep-like fatty aroma	1.05 ± 0.98	8.67 ± 0.97	0.000
Peppery aroma	15.51 ± 0.70	14.86 ± 0.93	0.554
Sweet aroma	18.77 ± 0.42	14.14 ± 0.81	0.000
Herbaceous aroma	0.86 ± 0.12	3.99 ± 1.02	0.000
**FLAVOUR**			
Overall flavour intensity	63.03 ± 1.60	54.79 ± 1.45	0.000
Smoky flavour	57.27 ± 1.52	45.92 ± 2.43	0.000
Woody flavour	18.22 ± 0.96	12.75 ± 0.97	0.000
Cured pork flavour	34.54 ± 1.88	22.92 ± 1.50	0.000
Fermented sour flavour	23.05 ± 0.71	16.17 ± 1.74	0.000
Pork fat flavour	18.27 ± 2.05	3.98 ± 1.87	0.000
Mutton flavour	1.32 ± 1.47	21.41 ± 2.18	0.000
Sheep-like fatty flavour	1.52 ± 0.64	15.47 ± 1.70	0.000
Peppery flavour	17.15 ± 0.99	15.86 ± 0.87	0.176
Herbaceous flavour	2.17 ± 0.59	8.32 ± 1.22	0.000
Salty taste	19.72 ± 0.75	18.58 ± 0.74	0.031
Sweet taste	19.61 ± 1.27	16.13 ± 1.37	0.002
Fatty mouthfeel	16.17 ± 1.07	22.03 ± 1.14	0.000
**APPEARANCE**			
Red/brown colour intensity	52.89 ± 2.73	47.85 ± 4.46	0.027
Fatty/Oily/Shininess	48.95 ± 3.99	47.84 ± 4.65	0.991
Perceived Percentage fat	46.09 ± 1.00	44.57 ± 1.72	0.642
**TEXTURE**			
First bite	29.91 ± 1.63	28.67 ± 2.00	0.218
Sustained juiciness	45.16 ± 1.24	50.74 ± 2.46	0.014
Chewiness	25.42 ± 0.70	22.29 ± 0.89	0.002
Residue	23.12 ± 1.54	18.21 ± 1.08	0.000

All data are expressed as mean ± SE (n = 12).

**Table 6:** Least significant means (±SE) of the preference and factors influencing the purchase of game meat products within the sample population.

Product	Least Significant Mean	Factor	Least Significant Mean
Biltong	7.7 ^a^ ± 1.69	Availability	6.2 ^c,d^ ± 1.91
Cabanossi	6.3 ^d^ ± 2.01	Fat content	6.5 ^b,c^ ± 1.87
Droëwors	6.9 ^b^ ± 1.84	Origin	5.5 ^e^ ± 2.52
Fresh meat	6.7 ^b,c^ ± 1.89	Price	7.2 ^a^ ± 1.77
Fresh/raw sausage	6.6 ^b^ ± 1.81	Safety	6.7 ^b^ ± 2.16
Salami	6.3 ^c,d^ ± 1.94	Species	5.8 ^d,e^ ± 2.26

^a–e^ Means with different superscripts between columns are significantly different.

**Table 7:** Least significant means for overall acceptance of cabanossi made with either pork backfat (PF) or sheep tail/backfat (SF).

Attribute	PF	SF
Appearance	6.75 ^a^ ± 1.38	6.27 ^b^ ± 1.61
Taste	6.75 ^a^ ± 1.64	6.12 ^b^ ± 1.81

^a,b^ Means with different superscripts between columns are significantly different. All data are expressed as mean ± SE.
